# Security of a kind of quantum secret sharing with entangled states

**DOI:** 10.1038/s41598-017-02543-0

**Published:** 2017-05-30

**Authors:** Tian-Yin Wang, Ying-Zhao Liu, Chun-Yan Wei, Xiao-Qiu Cai, Jian-Feng Ma

**Affiliations:** 1 School of Computer Science and Technology, Xidian University, Xian, 710071 China; 2 School of Mathematical Science, Luoyang Normal University, Luoyang, 471934 China; 3 School of Network and Information Security, Xidian University, Xian, 710071 China

## Abstract

We present a new collusion attack to a kind of quantum secret sharing schemes with entangled states. Using this attack, an unauthorized set of agents can gain access to the shared secret without the others’ cooperation. Furthermore, we establish a general model for this kind of quantum secret sharing schemes and then give some necessary conditions to design a secure quantum secret sharing scheme under this model.

## Introduction

The concept of secret sharing schemes was firstly introduced by Shamir^1^ and Blakely^2^, respectively, in which a secret *S* is divided into *n* pieces in such a way that *S* can be easily reconstructed from any *k* pieces, but even complete knowledge of *k* − 1 pieces reveals absolutely no information about *S*. The unique technique of secret sharing enables the construction of robust key management schemes or any other cryptographic schemes that can function securely and reliably even when misfortunes destroy half the pieces and security breaches expose all but one of the remaining pieces^1^.

In contrast to classical secret sharing, the security of quantum secret sharing (QSS) is based on the fundamental principles of quantum physics, which allows agents (holders of the shared secret) to share a secret securely even in the presence of an opponent Eve with unlimited computing ability^3^. Owning to the advantage of unconditional security, QSS has attracted much attention and a lot of schemes have been presented both in theoretical and experimental aspects^4–12^.

Although an opponent Eve must compromise at least *k* agents to learn the shared secret, and corrupt more than *n* − *k* shares to destroy the information in a (*k*, *n*) threshold sharing secret scheme, she has the entire life-time of the secret to mount these attacks. Gradual and instantaneous break-ins into a subset of agents over a long period of time may be feasible for her. Accordingly, the protection provided by traditional secret sharing may be not sufficient. A natural defense is to periodically refresh the secrets, but it is not always possible in some cases such as cryptographic master key and proprietary trade-secret information. As a result, what is actually required to protect the secret of the information is to periodically renew the shares without changing the secret, in such a way that any information learned by Eve about individual shares becomes obsolete after renewing the shares. This is so-called proactive secret sharing, which was firstly introduced by Herzberg *et al*.^13^ So far, many proposals for proactive secret sharing have been given in classical cryptography^14, 15^.

Based on two-step quantum secure direct communication (QSDC)^16^, a proactive QSS scheme (named QD-scheme hereafter) was proposed recently^17^, in which a dealer Alice prepares Einstein-Podolsky-Rosen (EPR) pairs and then sends all the second particles to every agent in sequence, and the agents code their shares on these particles with four local unitary operations. However, Gao and Wang show that the QD-scheme is not secure in the sense that dishonest participants may collaborate to eavesdrop the secret of the dealer without introducing any error^18^.

In this paper, we take the QD-scheme as an example and present a new collusion attack to this kind of QSS scheme based on QSDC, whereby an unauthorized set (the first agent and the last one) can gain access to the dealer’s secret without the others’ cooperation if they collude with each other. Then we establish a general model for this kind of QSS schemes. Finally, we give some necessary conditions to design a secure QSS scheme under this model.

## Results

### The QD-scheme

In the QD-scheme, *n* + 1 participants, i.e., the dealer Alice and *n* agents Bob_1_, Bob_2_, …, Bob_*n*_ are involved. Suppose that Alice wants to share a secret *S* among the *n* agents. The QD-scheme includes the following three phases^17^.

## Distribution

(1) Alice generates *m* EPR pairs $|{\rm{\Psi }}\rangle ={\otimes }_{i=1}^{m}{|{\rm{\Psi }}\rangle }_{{x}_{i},{y}_{i}}$, ${x}_{i},{y}_{i}\in \mathrm{\{0},\mathrm{1\},}\,i=1,\mathrm{2,}\ldots ,m$, each is randomly in one of the four Bell states:

$$\begin{array}{rcl}|{{\rm{\Psi }}}_{00}\rangle  & = & \frac{1}{\sqrt{2}}(|00\rangle +|11\rangle ),|{{\rm{\Psi }}}_{01}\rangle =\frac{1}{\sqrt{2}}(|10\rangle -|11\rangle ),\\ |{{\rm{\Psi }}}_{10}\rangle  & = & \frac{1}{\sqrt{2}}(|10\rangle +|01\rangle ),|{{\rm{\Psi }}}_{11}\rangle =\frac{1}{\sqrt{2}}(|10\rangle -|01\rangle ),\end{array}$$

hereafter the first particles of all EPR pairs $|{\rm{\Psi }}\rangle$ are called [*x*] sequence and the second are called [*y*] sequence. Then she prepares some decoy particles $|{\rm{0}}\rangle ,|{\rm{1}}\rangle ,|+\rangle =\frac{1}{\sqrt{2}}(|{\rm{0}}\rangle +|{\rm{1}}\rangle ),|-\rangle =\frac{1}{\sqrt{2}}(|{\rm{0}}\rangle -|{\rm{1}}\rangle )$ (BB84 particles) and inserts them into the [*y*] sequence. After that, she sends the [*y*] sequence to Bob_1_, and keeps a record of the insertion positions and initial states of the decoy particles.

(2) After confirming that Bob_1_ has received the [*y*] sequence, Alice publicly announces the position of the decoy particles and asks Bob_1_ to measure these particles with the base $Z=\{|{\rm{0}}\rangle ,|{\rm{1}}\rangle \}$ or $X=\{|+\rangle ,|-\rangle \}$ according to their bases and publish his measurement results. Then Alice computes the error rate through comparing the measurement results to the initial states. If the error rate exceeds the preset threshold, she asks Bob_1_ to abort the process and start a new one. Otherwise, they continue to perform the protocol.

(3) Bob_1_ randomly chooses a binary number ${K}^{1}=({u}_{1}^{1},{v}_{1}^{1},\ldots ,{u}_{m}^{1},{v}_{m}^{1})$ as his private key and then performs the unitary operation ${U}_{{u}_{i}^{1},{v}_{i}^{1}}$ on the *i* th particle in the [*y*] sequence, *i* = 1, 2, …, *m*, where ${U}_{{u}_{i}^{1},{v}_{i}^{1}}$ is one of the Pauli operators:

$$\begin{array}{rcl}{U}_{00} & = & I=|0\rangle \langle 0|+|1\rangle \langle 1|,{U}_{01}={\sigma }_{z}=|0\rangle \langle 0|-|1\rangle \langle 1|,\\ {U}_{10} & = & {\sigma }_{x}=|0\rangle \langle 1|+|1\rangle \langle 0|,{U}_{11}=i{\sigma }_{y}=|0\rangle \langle 1|-|1\rangle \langle 0|.\end{array}$$

Note that the [*y*] sequence is denoted as [*y*_1_] sequence after Bob_1_’s operation hereafter. Then he prepares some BB84 particles and inserts them into the [*y*_1_] sequence. After that, he sends the [*y*_1_] sequence to Bob_2_.

(4) Bob_2_ does the similar actions as Bob_1_. This process is continued until Bob_*n*_ sends [*y*_*n*_] sequence to Alice.

(5) After confirming the security of the [*y*_*n*_] sequence, Alice performs a Bell measurement on each EPR pair of the sequence $|{\rm{\Psi }}^{\prime} \rangle ={\otimes }_{i=1}^{m}{|{\rm{\Psi }}\rangle }_{{x}_{i}^{{\prime} },{y}_{i}^{{\prime} }}$, where $|{\rm{\Psi }}^{\prime} \rangle$ is the evolution of $|{\rm{\Psi }}\rangle$ after all the agents’ operations. According to the measurement outcome, she gets the secret *S* by computing

1 $$S=({x}_{1},{y}_{1},\ldots ,{x}_{m},{y}_{m})\oplus ({x}_{1}^{{\prime} },{y}_{1}^{{\prime} }\mathrm{,...,}{\,x}_{m}^{{\prime} },{y}_{m}^{{\prime} }),$$

hereafter ⊕ denotes the bitwise exclusive OR.

### Updating

(I) In the first updating period, Bob_1_ randomly generates *m* EPR pairs $|\overline{{\rm{\Psi }}}\rangle ={\otimes }_{i=1}^{m}{|{\rm{\Psi }}\rangle }_{\overline{{x}_{i}},\overline{{y}_{i}}}$, $\overline{{x}_{i}},\overline{{y}_{i}}\in \mathrm{\{0,}\,\mathrm{1\},}\,i=1,\mathrm{2,}\ldots ,m$, the first and the second particles of them are called $[\overline{x}]$ sequence and $[\overline{{y}_{1}}]$ sequence, respectively. Then Bob_1_ sends the $[\overline{{y}_{1}}]$ sequence to Bob_2_ after similarly processing in the distribution phase.

(II) Bob_2_ randomly chooses a binary number ${C}^{2}=({a}_{1}^{2},{b}_{1}^{2},\ldots ,{a}_{m}^{2},{b}_{m}^{2})$ and performs the unitary operation ${U}_{{a}_{i}^{2},{b}_{i}^{2}}$ on the *i* th particle in the $[\overline{{y}_{1}}]$ sequence, *i* = 1, 2, …, *m*. Then Bob_2_ updates his key by computing *K* ^2^ ⊕ *C* ^2^.

(III) The $[\overline{{y}_{1}}]$ sequence is denoted as $[\overline{{y}_{2}}]$ sequence after Bob_2_’s actions. Bob_2_ sends the $[\overline{{y}_{2}}]$ sequence to Bob_3_, and Bob_3_ performs the similar operation on the $[\overline{{y}_{2}}]$ sequence as Bob_2_. This process is continued until Bob_*n*_ sends $[\overline{{y}_{n}}]$ sequence to Bob_1_.

(IV) After confirming the security of the $[\overline{{y}_{n}}]$ sequence, Bob_1_ performs a Bell measurement on each EPR pair of $|\overline{{\rm{\Psi }}^{\prime} }\rangle ={\otimes }_{i=1}^{m}{|{\rm{\Psi }}\rangle }_{\overline{{x}_{i}^{{\prime} }},\overline{{y}_{i}^{{\prime} }}}$, where $|\overline{{\rm{\Psi }}^{\prime} }\rangle$ is the evolution of $|\overline{{\rm{\Psi }}}\rangle$ after the agents’ operations. After that, Bob_1_ updates his key as *K* ^1^ ⊕ *C* ^1^, where

2 $${C}^{1}=(\overline{{x}_{1}},\overline{{y}_{1}},\ldots ,\overline{{x}_{m}},\overline{{y}_{m}})\oplus (\overline{{x}_{1}^{{\prime} }},\overline{{y}_{1}^{{\prime} }}\mathrm{,...,}\overline{{x}_{m}^{{\prime} }},\overline{{y}_{m}^{{\prime} }})\mathrm{.}$$

(V) After the above steps, the first updating period is over. When the second updating period starts, Bob_2_ does the similar actions as Bob_1_. The other updating is performed periodically in the same way.

### Recovery

To recover the secret *S*, a trusted DC (designed combiner by the agents) is needed.

(A) DC randomly generates *m* EPR pairs $|\widehat{{\rm{\Psi }}}\rangle ={\otimes }_{i=1}^{m}{|\widehat{{\rm{\Psi }}}\rangle }_{\widehat{{x}_{i}},\widehat{{y}_{i}}}$.

(B) The $[\widehat{y}]$ sequence is sent to each agent Bob_*j*_ (*j* = 1, 2, …, *n*) in turn. Bob_*j*_ performs the unitary operation ${U}_{{u}_{i}^{j},{v}_{i}^{j}}$ on the *i* th (*i* = 1, 2, …, *m*) particle in the $[\widehat{y}]$ sequence according to his key ${K}^{j}=({u}_{1}^{j},{v}_{1}^{j},\ldots ,{u}_{m}^{j},{v}_{m}^{j})$.

(C) After finishing his operations, Bob_*n*_ sends the $[\widehat{y}]$ sequence to DC.

(D) When receiving the $[\widehat{y}]$ sequence, DC performs a Bell measurement on each EPR pair of $|\widehat{{\rm{\Psi }}^{\prime} }\rangle ={\otimes }_{i=1}^{m}{|{\rm{\Psi }}\rangle }_{\widehat{{x}_{i}^{{\prime} }},\widehat{{y}_{i}^{{\prime} }}}$, where $|\widehat{{\rm{\Psi }}^{\prime} }\rangle$ is the new state of $|\widehat{{\rm{\Psi }}}\rangle$ after the agents’ operations. Then DC recovers the secret *S* by computing

3 $$S=(\widehat{{x}_{1}},\widehat{{y}_{1}},\ldots ,\widehat{{x}_{m}},\widehat{{y}_{m}})\oplus (\widehat{{x}_{1}^{{\prime} }},\widehat{{y}_{1}^{{\prime} }},\ldots ,\widehat{{x}_{m}^{{\prime} }},\widehat{{y}_{m}^{{\prime} }})\mathrm{.}$$

By the property of the EPR pairs and four encoding operations, we can know $|{\rm{\Psi }}^{\prime} \rangle ={\otimes }_{i=1}^{m}{|{\rm{\Psi }}\rangle }_{{x}_{i}^{{\prime} },{y}_{i}^{{\prime} }}={\otimes }_{i=1}^{m}{|{\rm{\Psi }}\rangle }_{{x}_{i}\oplus {u}_{i}^{1}\oplus \cdots \oplus {u}_{i}^{n},{y}_{i}\oplus {v}_{i}^{1}\oplus \cdots \oplus {v}_{i}^{n}}$, which means ${x}_{i}^{{\prime} }={x}_{i}\oplus {u}_{i}^{1}\oplus \cdots \oplus {u}_{i}^{n}$ and ${y}_{i}^{{\prime} }={y}_{i}\oplus {v}_{i}^{1}\oplus \cdots \oplus {v}_{i}^{n}$. So, $S=({x}_{1},{y}_{1},\ldots ,{x}_{m},{y}_{m})\oplus ({x}_{1}^{{\prime} },{y}_{1}^{{\prime} },\ldots ,{x}_{m}^{{\prime} },{y}_{m}^{{\prime} })={K}^{1}\oplus {K}^{2}\oplus \cdots \oplus {K}^{n}$. Similarly, we can get ${C}^{1}=(\overline{{x}_{1}},\overline{{y}_{1}},\ldots ,\overline{{x}_{m}},\overline{{y}_{m}})\oplus (\overline{{x}_{1}^{{\prime} }},\overline{{y}_{1}^{{\prime} }},\ldots ,\overline{{x}_{m}^{{\prime} }},\overline{{y}_{m}^{{\prime} }})={C}^{2}\oplus \cdots \oplus {C}^{n}$. Clearly, after the first updating period of keys, the shared secret is ${K}^{1}\oplus {C}^{1}\oplus {K}^{2}\oplus {C}^{2}\oplus \cdots \oplus {K}^{n}\oplus {C}^{n}$ = ${K}^{1}\oplus {K}^{2}\oplus \cdots \oplus {K}^{n}=S$. The other updating periods of keys are similar to the first, and thus the shared secret *S* is not changed after the updating of keys. Therefore, the recovered secret by equation (3) is $(\widehat{{x}_{1}},\widehat{{y}_{1}},\ldots ,\widehat{{x}_{m}},\widehat{{y}_{m}})\oplus (\widehat{{x}_{1}^{{\prime} }},\widehat{{y}_{1}^{{\prime} }},\ldots ,\widehat{{x}_{m}^{{\prime} }},\widehat{{y}_{m}^{{\prime} }})={K}^{1}\oplus {K}^{2}\oplus \cdots \oplus {K}^{n}=S$ by deducting.

### The collusion scheme

As we know, the security of QSS requires that only an authorized set of agents can recover the secret *S* distributed by the dealer, but any unauthorized set of agents can gain access to nothing about it. Consequently, the main goal for the security of QSS is to prevent dishonest agents from deceiving. Nevertheless, the dishonest agents have a lot of advantages in contrast to outside opponents. On the one hand, they know partial information legally. On the other hand, they can tell a lie in the process of eavesdropping check to avoid introducing errors. Therefore, it is more complicated to analyse the security of QSS schemes compared with two-party cryptographic schemes^19–21^.

From the QD-scheme, it can be seen that the distribution phase, the updating phase and the recovery phase are very similar, all of them are based on QSDC. Here we take the distribution phase as an example to show its insecurity. In the distribution phase, the [*y*] sequence prepared by Alice is transferred among *n* agents Bob_1_, Bob_2_,…, Bob_*n*_ in turn, and when it is sent to an agent Bob_*j*_ (*j* = 1, 2, …, *n*), Bob_*j*_ encodes his share ${K}^{j}=({u}_{1}^{j},{v}_{1}^{j},\ldots ,{u}_{m}^{j},{v}_{m}^{j})$ to the [*y*] sequence by performing pauli operations ${U}_{{u}_{i}^{j},{v}_{i}^{j}}$, *i* = 1, 2, …, *m*. Although each agent Bob_*j*_ (*j* = 1, 2, …, *n*) checks the security of quantum channel between him and the previous agent Bob_*j*−1_, and Alice checks the security of quantum channel between her and the agent Bob_*n*_, there is also a chance for dishonest agents to deceive. Specifically, the first agent Bob_1_ and the last agent Bob_*n*_, an unauthorized set of agents, can gain access to the shared secret *S* without the cooperation of any other agent if they collude with each other by the following collusion attack.

(i) In the distribution phase, Bob_1_ prepares *m* EPR pairs $|{\rm{\Psi }}^{\prime\prime} \rangle ={\otimes }_{i=1}^{m}{|{\rm{\Psi }}\rangle }_{{x}_{i}^{{\prime\prime} },{y}_{i}^{{\prime\prime} }}$ in advance, ${x}_{i}^{{\prime\prime} },{y}_{i}^{{\prime\prime} }\in \mathrm{\{0},\mathrm{1\},}{|{\rm{\Psi }}\rangle }_{{x}_{i}^{{\prime\prime} },{y}_{i}^{{\prime\prime} }}\in \{|{{\rm{\Psi }}}_{00}\rangle ,|{{\rm{\Psi }}}_{01}\rangle ,|{{\rm{\Psi }}}_{10}\rangle ,|{{\rm{\Psi }}}_{11}\rangle \},i=1,\mathrm{2,}\ldots ,m$. The first particles of all EPR pairs $|{\rm{\Psi }}^{\prime\prime} \rangle$ are called [*x*″] sequence and the second are called [*y*″] sequence. Then he sends the initial Bell state information $({x}_{1}^{{\prime\prime} },{y}_{1}^{{\prime\prime} },\ldots ,{x}_{m}^{{\prime\prime} },{y}_{m}^{{\prime\prime} })$ and the [*x*″] sequence to Bob_*n*_.

(ii) As does in Steps (2) and (3), Bob_1_ performs his actions faithfully except that he inserts BB84 decoy particles into the [*y*″] sequence and sends it to Bob_2_ instead of the [*y*_1_] sequence, and sends the real [*y*_1_] sequence to Bob_*n*_.

(iii) When Bob_*n*_ receiving the fake [*y*_*n*−1_] sequence from Bob_*n*−1_, i.e., [*y*″] sequence, he performs a Bell measurement on each EPR pair of $|{\rm{\Psi }}^{\prime\prime\prime} \rangle ={\otimes }_{i=1}^{m}{|{\rm{\Psi }}\rangle }_{{x}_{i}^{{\prime\prime\prime} },{y}_{i}^{{\prime\prime\prime} }}$ after checking the security of quantum channel between him and Bob_*n*−1_, where $|{\rm{\Psi }}^{\prime\prime\prime} \rangle$ is the evolution of $|{\rm{\Psi }}^{\prime\prime} \rangle$ after the agents’ operations.

(iv) As does in Step (4), Bob_*n*_ randomly chooses a binary number ${K}^{n}=({u}_{1}^{n},{v}_{1}^{n},\ldots ,{u}_{m}^{n},{v}_{m}^{n})$ as his private key. Then he computes

4 $$\begin{array}{rcl}S^{\prime\prime\prime\prime}  & = & ({x}_{1}^{{\prime\prime} },{y}_{1}^{{\prime\prime} },\ldots ,{x}_{m}^{{\prime\prime} },{y}_{m}^{{\prime\prime} })\oplus ({x}_{1}^{{\prime\prime\prime} },{y}_{1}^{{\prime\prime\prime} },\ldots ,{x}_{m}^{{\prime\prime\prime} },{y}_{m}^{{\prime\prime\prime} })\\  & = & ({x}_{1}^{{\prime\prime} ^{\prime\prime} },{y}_{1}^{{\prime\prime} ^{\prime\prime} },\ldots ,{x}_{m}^{{\prime\prime} ^{\prime\prime} },{y}_{m}^{{\prime\prime} ^{\prime\prime} })\mathrm{.}\end{array}$$

After that, he performs the operation ${U}_{{u}_{i}^{n},{v}_{i}^{n}}{U}_{{x}_{i}^{{\prime\prime} ^{\prime\prime} },{y}_{i}^{{\prime\prime} ^{\prime\prime} }}$ on the *i* th particle in the real [*y*_1_] sequence received from Bob_1_, *i* = 1, 2, …, *m*.

(v) As does in Step (4), after inserting BB84 decoy particles into [*y*_*n*_] (the real [*y*_1_] sequence after Bob_*n*_’s operation), Bob_*n*_ sends it to Alice.

(vi) After the completion of distribution, Bob_1_ and Bob_*n*_ can recover the shared secret *S* at any time by computing

5 $$\tilde{S}={K}^{1}\oplus S^{\prime\prime\prime\prime} \oplus {K}^{n}\mathrm{.}$$

Now let us prove the effectiveness of joint attack. Firstly, it is evident that this deception introduces no error and therefore cannot be detected in the process of eavesdropping check from the above attack. Secondly, the EPR pairs generated by Bob_1_ in Step (i) are $|{\rm{\Psi }}^{\prime\prime} \rangle ={\otimes }_{i=1}^{m}{|{\rm{\Psi }}\rangle }_{{x}_{i}^{{\prime\prime} },{y}_{i}^{{\prime\prime} }}$, and the private keys generated by Bob_2_, Bob_3_,…, Bob_*n*−1_ are also ${K}^{2}=({u}_{1}^{2},{v}_{1}^{2},\ldots ,{u}_{m}^{2},{v}_{m}^{2})$, ${K}^{3}=({u}_{1}^{3},{v}_{1}^{3},\ldots ,{u}_{m}^{3},{v}_{m}^{3})$, …, ${K}^{n-1}=({u}_{1}^{n-1},{v}_{1}^{n-1},\ldots ,{u}_{m}^{n-1},{v}_{m}^{n-1})$, respectively. By the property of EPR pairs and Pauli operators, the EPR pairs $|{\rm{\Psi }}^{\prime\prime} \rangle$ will evolve in the state

6 $$\begin{array}{rcl}|{\rm{\Psi }}^{\prime\prime\prime} \rangle  & = & {\otimes }_{i=1}^{m}{|{\rm{\Psi }}\rangle }_{{x}_{i}^{{\prime\prime\prime} },{y}_{i}^{{\prime\prime\prime} }}\\  & = & {\otimes }_{i=1}^{m}{|{\rm{\Psi }}\rangle }_{{x}_{i}^{{\prime\prime} }\oplus {u}_{1}^{2}\oplus {u}_{1}^{3}\oplus \cdots \oplus {u}_{1}^{n-1},{y}_{i}^{{\prime\prime} }\oplus {v}_{1}^{2}\oplus {v}_{1}^{3}\oplus \cdots \oplus {v}_{1}^{n-1}}\end{array}$$

after the unitary operations of Bob_2_, Bob_3_, …, Bob_*n*−1_. Therefore, we can get

7 $$S^{\prime\prime\prime\prime} ={K}^{2}\oplus {K}^{3}\oplus \cdots \oplus {K}^{n-1},$$

which means

8 $$\tilde{S}={K}^{1}\oplus {K}^{2}\oplus \cdots \oplus {K}^{n}\mathrm{.}$$

Finally, after the unitary operations of Bob_1_ and Bob_*n*_, the EPR pairs $|{\rm{\Psi }}\rangle ={\otimes }_{i=1}^{m}{|{\rm{\Psi }}\rangle }_{{x}_{i},{y}_{i}}$ prepared by Alice will evolve in the state

9 $$\begin{array}{rcl}|{\rm{\Psi }}^{\prime} \rangle  & = & {\otimes }_{i=1}^{m}{|{\rm{\Psi }}\rangle }_{{x}_{i}^{{\prime} },{y}_{i}^{{\prime} }}\\  & = & {\otimes }_{i=1}^{m}{|{\rm{\Psi }}\rangle }_{{x}_{i}\oplus {u}_{1}^{1}\oplus {x}_{i}^{{\prime\prime} ^{\prime\prime} }\oplus {u}_{1}^{n},{y}_{i}\oplus {v}_{1}^{1}\oplus {y}_{i}^{{\prime\prime} ^{\prime\prime} }\oplus {v}_{1}^{n}}\\  & = & {\otimes }_{i=1}^{m}{|{\rm{\Psi }}\rangle }_{{x}_{i}\oplus {u}_{1}^{1}\oplus {u}_{1}^{2}\oplus \cdots \oplus {u}_{1}^{n},{y}_{i}\oplus {v}_{1}^{1}\oplus {v}_{1}^{2}\oplus \cdots \oplus {v}_{1}^{n}}\end{array}$$

which means that the secret *S* also satisfies

10 $$S={K}^{1}\oplus {K}^{2}\oplus \cdots \oplus {K}^{n}\mathrm{.}$$

Obviously, $\tilde{S}=S$. Additionally, as shown in the QD-scheme^17^, the shared secret *S* is not changed after the updating of keys.

As a result, Bob_1_ and Bob_*n*_ can gain access to the shared secret *S* at any time without the others’ cooperation if they collude with each other, which is in conflict with the security requirement of QSS that only an authorized set of agents can recover the secret *S*, but the unauthorized set of agents can gain access to nothing about it.

Noted that Bob_1_ and Bob_*n*_ also can directly gain access to the shared secret *S* in the recovery phase if they collude with each other by the similar joint attack.

### The proposed model

In this section, let us give a general model for this kind of QSS schemes based on QSDC. Let *k* be the security parameter. The general procedure for this kind of QSS can be rephrased in the following.

1) Alice prepares *m* quantum states $|\varphi \rangle ={\otimes }_{i=1}^{m}{|\varphi \rangle }_{i}$ (two-particle or multi-particle entangled states). Then she takes one particle from each entangled states ${|\varphi \rangle }_{i}$ to form a travel sequence (named *T*-sequence hereafter). After that, she prepares 2*k* decoy particles and inserts them into the *T*-sequence before sending it to Bob_1_.

2) When receiving the *T*-sequence, Bob_1_ firstly ascertains whether each particle in the *T*-sequence is sure a single one or not by the similar methods in refs 22–24. If it is so, Alice tells Bob_1_ the initial states and positions of *k* decoy particles and then Bob_1_ checks whether the *T*-sequence is secure or not by the measurement outcomes on them. If it is secure, for each particle in the *T*-sequence, Bob_1_ chooses two unitary operations *U*, *U*′ and then performs the operation *U*′*U* on it, where *U* is chosen from a set $\tilde{U}$ according to his sub-secret *K* ^1^ and is used to encode his sub-secret, *U*′ is randomly chosen from a set $\tilde{U^{\prime} }$ and is used to encrypt his sub-secret. After that, he also prepares *k* decoy particles and inserts them into the *T*-sequence before sending it to Bob_2_. In other cases, he aborts the protocol and asks Alice to restart.

3) Bob_2_ performs the similar actions as Bob_1_ does in Step 2) after receiving the *T*-sequence. This process is repeated until Bob_*n*_ sends the *T*-sequence to Alice.

4) When receiving the *T*-sequence, Alice also firstly ascertains whether each of them is sure a single particle or not. If it is so, she announces the remaining *k* decoy particles’ positions to the agents and requires them to send their unitary operations *U*′*U* performed on these particles to her. Then she judges whether the *T*-sequence is attacked or not by the measurement outcomes on the *k* decoy particles. If it is secure, she requires all agents to send her their encryption operations *U*′ and then she performs a projective measurement on each entangled states ${|\varphi \rangle }_{i}$, *i* = 1, 2, …, *m*. According to the measurement outcomes and initial states, she can obtain the secret $S={K}^{1}\oplus {K}^{2}\oplus \cdots \oplus {K}^{n}$. In other cases, she aborts the protocol.

By running this program, Alice makes *n* agents share a secret *S* that can be reconstructed if and only if they cooperate together.

### The proposed conditions

Now let us study the necessary conditions to design a secure QSS scheme under this model. For QSS, the security mainly includes two aspects: the agents’ encoding operations (sub-secrets) and the shared secret *S*.

Firstly, let us analyse the conditions that nobody can obtain a agent’s sub-secret except himself. To get an agent Bob_*i*_’s sub-secret *K* ^*i*^, there are generally three ways for an opponent Eve: one is intercepting the *T*-sequence and then learning some information by directly measuring each particle in the *T*-sequence. The second is sending fake particles to Bob_*i*_ as the *T*-sequence and then intercepting them when they are sent to Bob_*i*+1_ by Bob_*i*_. After that, Eve tries to learn some information by measuring these fake particles later. The last is sending multi-particle signal to Bob_*i*_, i.e., Trojan horse attack: Eve inserts one or multi spy particles, an invisible particle, or a delay one in each particle of the *T*-sequence when it is sent to Bob_*i*_, and captures the spy particles when they are sent to the next agent Bob_*i*+1_ and gets some information by measuring them later. This kind of attacks were introduced in 2005 by Deng *et al*.^22^ and have been used to break through a lot of cryptographic schemes^23, 24^, and therefore we must seriously consider how to deal with them here. Let us analyse whether it is feasible or not by the first way, it can be seen from the proposed model that nobody knows the initial state of ${|\varphi \rangle }_{i}$ except Alice. In addition, Eve only has one particle of each entangled state ${|\varphi \rangle }_{i}$. Accordingly, she can learn no information on Bob_*i*_’s encoding operation *U* according to the principle of quantum measurement, which means that nobody can know an agent’s sub-secret by this way. If Eve wants to steal Bob_*i*_’s sub-secret *K* ^*i*^ by the second way, she must escape the security check on the *T*-sequence between Bob_*i*_ and Bob_*i*−1_ firstly. It is impossible for an outside opponent Eve to do that except with exponentially small probability, but it is not a problem for an inside opponent Bob_*i*−1_. Nevertheless, if Bob_*i*−1_ wants to steal Bob_*i*_’s sub-secret *K* ^*i*^ by directly measuring these fake particles, he must have the ability to discriminate the encoding operation *U* from the set $\tilde{U}$ after the encrypting operation *U*′, which is equivalent to discriminate the unitary operation *U*′*U* is in which one of the sets $\tilde{U^{\prime} }U$, $U\in \tilde{U}$, where

11 $$\tilde{U^{\prime} }U=\{U^{\prime} U|U^{\prime} \in U^{\prime} \}.$$

Nevertheless, the unitary operation *U*′*U* is performed on a fake particle (a single particle or one qubit of an entangled state) only once, if the two sets $\tilde{U^{\prime} }$ and $\tilde{U}$ are selected properly, Bob_*i*−1_ will not discriminate the unitary operation *U*′*U* is in which one of the sets $\tilde{U^{\prime} }U$, $U\in \tilde{U}$ only by measuring the fake particle. To get rid of this restriction, Bob_*i*−1_ can measure these fake particles after Bob_*i*_ publishes his encryption operation *U*′ in Step 4), but it requires his deception must escape the security check between Bob_*i*+1_ and Bob_*i*_ in Step 3), and Alice’s security check in Step 4). Obviously, if Bob_*i*+1_ is also dishonest, that is he colludes with Bob_*i*−1_, in this case Bob_*i*−1_’s deception can easily escape the security check between Bob_*i*+1_ and Bob_*i*_. To escape Alice’s security check in Step 4), the teleportation attack was proposed in 2008^20, 25, 26^, but how to prevent this attack will be analysed in the following paragraph. To steal Bob_*i*_’s sub-secret by the last way, Eve’s deception must escape Bob_*i*_’s multi-particle signal check. Nevertheless, it is very difficult because this kind of attacks can be prevented by technical measures. Li *et al*.^23^ gave a way to filter out invisible photons. Specifically, Bob_*i*_ can add a filter in his laboratory first. All photon pulses should pass through his filter first. Only wavelengths close to the operating wavelength can be let in. Thus, Eve’s invisible photons can be filtered out by using the filter. Furthermore, if Eve’s spy photons cannot be filtered out, Deng *et al*.^22^ gave a feasible way to detect them. Specifically, Bob_*i*_ chooses some sample signals and splits them with a photon number splitter, and then measures the two signals with Z-basis or X-basis randomly. If both the measurements have an outcome, Bob_*i*_ can judge the quantum signal is a multi-photon signal. Therefore, if Bob_*i*_ has the ability of discriminating whether each quantum signal only contains a single particle, this way will not be feasible any longer.

Secondly, let us analyse the conditions that nobody can recover the shared secret *S* except that all the agents cooperate together. Since the shared secret is the module sum of the agents’ sub-secrets, i.e., $S={K}^{1}\oplus {K}^{2}\oplus \cdots \oplus {K}^{n}$, the conditions of protecting sub-secrets should be firstly satisfied to maintain its security. To gain access to the shared secret *S*, one possible way is stealing all the agents’ sub-secrets *K* ^1^, *K* ^2^, …, *K* ^*n*^, whereby the difficulties have been analysed in the above paragraph. Another possible way is using teleportation attack. The basic principle of this attack can be described as the following. In step 2), a dishonest agent (e.g., Bob_1_) sends *m* + *k* fake particles (each of them is one qubit of a Bell state) instead of the *T*-sequence to the next agent. At the same time, he stores the real *T*-sequence and the remaining *m* + *k* qubits of the Bell states in his quantum database. In step 4), when Alice announces the remaining *k* decoy particles’ positions, Bob_1_ performs a teleportation measurement on the corresponding original decoy particle and the remaining qubit of the corresponding Bell state. By this way, the state of the corresponding original decoy particle can be teleported to the fake one (i.e., the one qubit of the corresponding Bell state sent to Alice in the end) by the principle of teleportation except the lack of a unitary operation, and therefore the dishonest agent can successfully hide his replacing deception by sending the corresponding unitary operation to Alice. The condition to prevent this attack under single particle model has been deeply discussed in ref. 27. By similar analysis, we can find this condition is also suitable for this model. Specifically, the condition is $\overline{U}\, \nsubseteq \,\langle \tilde{U},\tilde{U}^{\prime} \rangle$, where $\overline{U}$ denotes a unitary operation set that consists of the unitary operations corresponding to the teleportation measurement outcomes, and $\langle \tilde{U},\tilde{U}^{\prime} \rangle$ represents a unitary operation set, which consists of all the elements in $\tilde{U}$ and $\tilde{U^{\prime} }$ and all the possible products of them.

Up to now, we have clarified the conditions to prevent all the present attacks under the proposed model, i.e., (i) the dealer Alice and every agent have the ability to discriminate whether each quantum signal only contains a single particle; (ii) the unitary operation *U*′*U* ($U\in \tilde{U},U^{\prime} \in \tilde{U^{\prime} }$) cannot be discriminated in the set $\tilde{U^{\prime} }U$ when it is performed only on a single particle or one qubit of any entangled state; (iii) $\overline{U}\, \nsubseteq \,\langle \tilde{U},\tilde{U}^{\prime} \rangle$.

## Discussion

Using the given conditions, we can judge whether a QSS scheme under the proposed model is secure or not, i.e., if a QSS scheme under the proposed model does not satisfy all the conditions i)-iii), this scheme must be not secure, e.g., the QD-scheme is vulnerable to a lot of attacks because it satisfies none of the conditions i), ii) and iii); otherwise, this scheme is immune to all the present attacks in the sense that these attacks will be detected by Alice in the process of eavesdropping detection with probability *p*. The probability *p* can be computed by the following equation

12 $$p=1-{\mathrm{(1}-{p}_{e})}^{k},$$

where *p*_*e*_ denotes the least probability that an opponent introduces an error when a decoy particle is checked. Assume a QSS scheme under the proposed model satisfies all the conditions i)-iii), the least probability *p*_*e*_ only depends on the set $\tilde{U^{\prime} }\tilde{U}$ since the multi-particle signal attack and the invisible particle attack have been excluded by the condition i), and thus the least probability *p*_*e*_ is no less than 1/*r* since at least one of the unitary operations corresponding to teleportation measurement cannot be properly announced by the condition iii), where *r* is the element number of the set $\overline{U}$.

From Eq. (12), it can be seen that *p* is exponentially close to 1 with the increase of the security parameter *k*, which means that the opponent’s attack will be detected by Alice with probability exponentially close to 1.

It is evident that if the opponent’s attack is detected by Alice, he/she will get no information on the shared secret *S*. Nevertheless, Alice cannot distinguish which one is the attacker when she finds that there is deceiving among the agents in the process of eavesdropping check, which will induce that a dishonest agent may like to take the risk to cheat, because if the cheating is not detected then he will be benefited, while even if it is detected, he will be not blamed by Alice. Furthermore, when *k* is very small, the dishonest agent may have a chance to escape Alice’s detection.

Using the given conditions, we also can judge whether a QSS scheme is not secure if it is similar to the present model, e.g., the QSS scheme in ref. 28 is not secure since it does not satisfy the condition (iii). Nevertheless, we cannot give a full classification on the security of previous schemes by the conditions (i)-(iii) because most of them are far different from the present model.
